# Acrylamide and Potential Risk of Diabetes Mellitus: Effects on Human Population, Glucose Metabolism and Beta-Cell Toxicity

**DOI:** 10.3390/ijms23116112

**Published:** 2022-05-30

**Authors:** Jelena Marković Filipović, Jelena Karan, Ivana Ivelja, Milica Matavulj, Milena Stošić

**Affiliations:** 1 Department of Biology and Ecology, Faculty of Sciences, University of Novi Sad, Trg Dositeja Obradovića 2, 21000 Novi Sad, Serbia; jelena.karan@dbe.uns.ac.rs (J.K.); ivana.ivelja@dbe.uns.ac.rs (I.I.); milica.matavulj@dbe.uns.ac.rs (M.M.); 2 Department of Environmental Engineering and Occupational Safety and Health, Faculty of Technical Science, University of Novi Sad, Trg Dositeja Obradovića 6, 21000 Novi Sad, Serbia; milenastosic@uns.ac.rs

**Keywords:** acrylamide, diabetes mellitus, beta-cell toxicity, glucose metabolism, oxidative stress, in vitro, in vivo, human epidemiological studies

## Abstract

Diabetes mellitus is a frequent endocrine disorder characterized by hyperglycemia. Acrylamide (AA) is food contaminant formed during the high-temperature processing of food rich in carbohydrates and low in proteins. Recent human epidemiological studies have shown a potential association between AA exposure and the prevalence of diabetes in the general population. In male rats, AA treatment promoted pancreatic islet remodeling, which was determined by alpha-cell expansion and beta-cell reduction, while in female rats AA caused hyperglycemia and histopathological changes in pancreatic islets. In vitro and in vivo rodent model systems have revealed that AA induces oxidative stress in beta cells and that AA impairs glucose metabolism and the insulin signaling pathway. Animal studies have shown that diabetic rodents are more sensitive to acrylamide and that AA aggravates the diabetic state. In this review, we provide an overview of human epidemiological studies that examined the relation between AA exposure and glucose disorders. In addition, the effects of AA treatment on pancreatic islet structure, beta-cell function and glucose metabolism in animal models are comprehensively analyzed with an emphasis on sex-related responses. Furthermore, oxidative stress as a putative mechanism of AA-induced toxicity in beta cells is explored. Finally, we discuss the effects of AA on diabetics in a rodent model system.

## 1. Introduction

**Figure 1:**
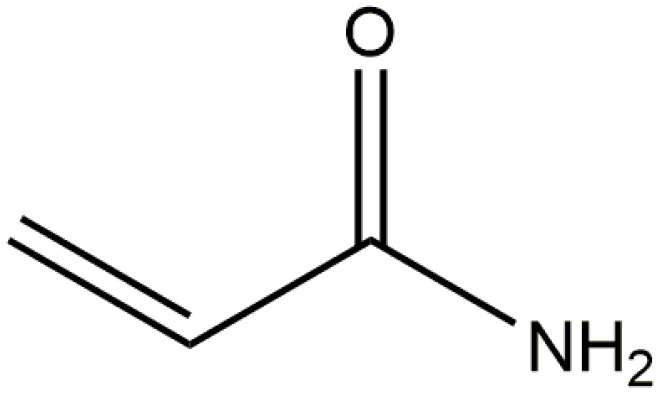
The chemical structure of acrylamide.

Acrylamide (AA) (2-propenamide, C_3_H_5_NO, CAS No. 79-06-1) is a hydrophilic molecule with a molar mass of 71.08 g/mol. It is a carbonyl derivative with two functional groups: an amide group and double bonds on the α and β carbon atoms, which are responsible for AA’s high reactivity (Figure 1) [1].

AA is a chemical substance that has a very diverse application in everyday life, especially in agriculture and industry. Primarily, AA is a building block of a very widely used polyacrylamide (PAA), a polymer considered to be a non-toxic additive. PAA has a widespread application because of its high viscosity and capability to reduce resistance and retain water [2]. One of the most common applications of PAA is in the treatment of wastewater and drinking water, where it is used as a flocculant [3,4]. PAA is also actively used as a soil conditioner in agriculture [2], in the process of paper production [5], in color synthesis and in the production of contact lenses as well as in construction as a grouting agent [6]. Given such widespread use of PAA, it is very important to emphasize that this polymer can undergo chemical, mechanical, thermal, photolytic and biological degradation, possibly leading to the release of AA in the environment, which increases the likelihood of exposure of living organisms to this toxic monomer [2]. Beside industrial use, AA was proven to be present in cigarette smoke, but more importantly, it was confirmed in various food items [7].

The main dietary sources of AA are food items obtained by processing food rich in carbohydrates and low in proteins [5]. The principal route of AA formation is a complex sequence of nonenzymatic reactions that occurs during the thermal treating of food called the Maillard reaction. These reactions occur between reducing sugars and asparagine [8]. Thus, AA in food does not form naturally and spontaneously, but it forms at high temperatures (>120 °C) in conditions when water is not used in its preparation, such as baking, toasting, frying or grilling [9]. Consequently, food items such as bread, French fries and potato chips are foods that often contain the highest concentrations of AA [10].

Acrylamide is not an emerging chemical that is newly present in our environment. Its use has been frequent and diverse for decades [11]. Humans are exposed to AA from a variety of different routes (oral, dermal or inhalational) and from diverse sources, which include diet, smoking, drinking water and occupational sources [5]. Acrylamide is a substance that is easily and quickly absorbed from water and food orally as well as by skin contact and inhalation [5]. After gavage administration of acrylamide aqueous solutions in rats [12] and mice [13], acrylamide was promptly absorbed. The assessment of acrylamide bioavailability is complex and difficult due to its fast metabolism to glycidmide in rodents. The half-life of free acrylamide in humans has been assessed to be only a few hours [9]. Acrylamide has a fast and total uptake via the gastrointestinal tract in rats, while intake via the dermal route is 25% of the used dose [14]. In the European Union, the minimum permitted presence of acrylamide in drinking water is determined by law and amounts to 0.1 μg/L [15]. Keeping these data in mind and assuming that the daily water intake is 2 L, a person weighing 70 kg ingests approximately 0.003 μg/kg body weight (μg/kg bw) of acrylamide daily [16]. Besides the dietary and drinking-water exposure, the intake of acrylamide in humans is often through tobacco smoke. It was proven that smoking is a more prominent source of acrylamide exposure compared to dietary intake [17]. Furthermore, because of its broad industrial use, professional exposure to acrylamide through dermal absorption (as a predominant route) or inhalation is also important [18]. The Joint Expert Committee on Food Additives reported that the main food items responsible for AA intake are potato crisps (6–46%), potato chips (16–30%), coffee (13–39%), pastries and sweet biscuits (10–20%) as well as bread (10–30%) [19]. Dietary intake has proven to be the primary source of AA exposure for the general nonsmoking population, with 38% of calorie intake provided by AA-containing food sources [5].

The dietary intake of AA can depend on different factors. The level of AA in food is in direct relation with the level of asparagine in each food item, with the temperature at which food is prepared and with the length of food treatment at a high temperature [11]. Worldwide, an average mean dietary AA intake is approximately 0.4 mg/kg b.w./d, and the average intake for a high-end consumer is around 1.0 mg/kg b.w./d [5]. In Europe, the mean dietary AA intake has been estimated to range from 0.4 to 1.9 μg/kg b.w./d, while in high-end consumers it can range up to 3.4 μg/kg b.w./d [20]. Children, including infants and toddlers, are undoubtedly the most exposed group due to their specific dietary pattern (children usually consume more starchy food items than adults) and also due to their lower body mass. The dietary intake in children was shown to range between 0.5 and 1.9 µg/kg b.w./day, while the 95th percentile ranged between 1.4 and 3.4 µg/kg b.w./day [21].

When absorbed, AA dissolves well in the blood and is evenly transferred to all organ systems. AA is metabolized in the body in two main metabolic pathways that are not equally represented. The first is far more common in humans and is based on the conjugation of AA with glutathione (GSH), which results in the formation of metabolites excreted by the urine [20]. The second metabolic pathway is the oxidative transformation of AA with cytochrome P450 2E1 (CYP2E1; EC 1.14.13) to epoxide 2,3-epoxypropane amide (glycidamide—GA), which is proven to have genotoxic properties [22]. Approximately 15% of internalized AA is epoxidated to GA, which, like AA, has electrophilic reactivity [23].

The covalent binding of AA and GA to the N-terminal valine residue of hemoglobin forms two types of hemoglobin adducts, N-(2-carbamoylethyl)valine (HbAA) and N-(2-carbamoyl-2-hydroxyethyl)valine (HbGA), respectively, which are subsequently used as biomarkers for long-term exposure monitoring [20,24]. In addition to these biomarkers in blood, AA and GA produce exposure biomarkers in urine with significantly shorter half-lives. Namely, both AA and GA react with glutathione (GSH)-forming GSH-conjugates that are altered to N-acetylcysteine thioethers: mercapturic acids (MA) N-acetyl-S-(carbamoylethyl)-l-cysteine (AAMA), N-acetyl-S-(1-carbamoyl-2-hydroxyethyl)-l-cysteine (GAMA) and N-acetyl-S-(2-carbamoylethyl)-l-cysteine-sulfoxide (AAMA-sul), which is a metabolite that is characteristic for humans [20].

The adverse effects of AA on living systems have already been proven in animal models as well as in humans and they include neurotoxicity, genotoxicity and carcinogenicity as well as reproductive and developmental toxicity in animal species [25,26,27,28,29]. The neurotoxicity of AA is the only toxic effect that is indisputably exhibited in both human occupational exposure and laboratory animals [9,30,31]. AA is classified as a probable human carcinogen (Group 2A) by the International Agency of Research on Cancer (IARC) due to its genotoxicity and carcinogenicity in rodents [32]. Additionally, AA can be classified into a group of substances that disrupt the endocrine system [26,33]. Although the average daily intake of AA is very low and of the order of parts per billion (ppb), the cumulative nature of AA toxicity is extremely important when it comes to human exposure to this substance since the exposure is chronic via different exposure routes. The low-dose chronic exposure to AA from the diet and its impact on human health are still not fully recognized. It is very hard to define a safe dietary AA dose that would pose an acceptable risk since the assessment of dietary AA exposure usually considers data from diet questionnaires, which do not appropriately reveal and represent lifetime exposure. Keeping in mind that endocrine-disrupting chemicals could generate biological changes at doses lower than the permitted dose, low-dose chronic AA exposure may pose a significant health problem.

AA leads to changes in oxidative stress parameters at both high and low doses [34]. AA-induced oxidative stress is characterized by an intensive lipid peroxidation process and elevated protein-carbonyl levels as well as a decreased intensity of enzymatic and non-enzymatic antioxidant processes [35].

Diabetes mellitus is a frequent endocrine disorder characterized by hyperglycemia, hyperinsulinemia, the dysfunction of insulin production and insulin sensitivity [36]. Type 1 diabetes is an autoimmune disease in which autoreactive T lymphocytes attack pancreatic beta cells, leading to insufficient insulin synthesis [37,38]. On the other hand, type 2 diabetes is a metabolic disorder in which pancreatic beta-cell failure is accompanied by insulin resistance, resulting in an organism that is resistant to the insulin it produces [37,38]. Without exaggeration, it can be considered that one of the biggest epidemics in the world is the diabetes mellitus epidemic. Although the term epidemic is usually related to an infectious disease, the high and promptly surging incidence of diabetes mellitus permits this description. Around 425 million adults are diagnosed with diabetes globally [23]. Some projections show that the prevalence of this disease is likely to increase to 629 million by 2045 [39]. A substantial number of epidemiological studies have offered clear indications that certain chemical substances, including food pollutants and food-processing-induced chemicals, are linked with a disruption in glucose metabolism, potentially leading to the onset of diabetes [23,40,41,42,43]. AA may be one of the chemicals that are a contributing factor in the prevalence of diabetes.

This review aims to investigate the available literature data regarding the effects of AA on pancreatic islet structure, beta-cell function and glucose metabolism as well as to analyze the possible mechanisms of AA-induced beta-cell toxicity, with an emphasis on oxidative stress. Moreover, the effects of AA on diabetics as a sensitive population are discussed. Finally, an overview of human epidemiological studies regarding the association between AA intake and the prevalence of diabetes mellitus is provided.

## 2. Potential Association between Acrylamide Intake and Diabetes Mellitus in Human Population

There are some human epidemiological studies that exhibit the relation between AA exposure and glucose metabolism disorders, including insulin-level variations [26]. Wang et al. [1] found a considerable positive dose-related link between AA exposure and increases in fasting plasma glucose and lipid peroxidation together with oxidative DNA damage in the general Chinese population. Additionally, the results of one pilot study indicated an increase in fasting plasma glucose after long-term consumption of potato-chips but with no statistical significance due to the small number of examined subjects [44]. Potato chips are one of the main sources of dietary AA [19]. Lin et al. [45] showed from survey data from the National Health and Nutrition Examination Survey (NHANES) that AA exposure was associated with a decreased insulin level and with the insulin resistance status in the American adult population. The study conducted by Yin et al. [23] estimated the link between AA serum biomarkers with the prevalence of diabetes mellitus in the general population. This study revealed that HbAA was in inverse proportion with the occurrence of diabetes mellitus, while the HbGA/HbAA biomarker was positively correlated with the risk of diabetes mellitus in the American adult population.

In addition, AA is linked with obesity, hyperlipidemia and metabolic syndrome [46,47]. There is a significant inverse correlation between HbAA and body mass index and a positive relationship between HbGA levels and obesity in adult populations [46,48]. The results of two independent longitudinal birth cohort studies from France and Norway that were conducted on children showed that exposure to elevated levels of AA during the prenatal period resulted in infants who were more likely to be born small for gestational age and become overweight at the age of 3 years [49,50]. In humans, HbAA adducts in the blood are related with abdominal obesity and excessive weight, but the mode of action by which acrylamide generates the differentiation of adipose tissue and obesity through lipid metabolism is uncertain [46,51].

Since potato products are one of the main sources of dietary AA [19], several studies dealt with the possible link between potato consumption and the occurrence of diabetes mellitus type 2 but without a distinctive consensus. While some studies reported a positive correlation between potato consumption and the prevalence of type 2 diabetes [47,52], Liu et al. [53] proposed an inverse correlation. Zhang et al. [47] demonstrated a significant positive relationship between elevated potato consumption and the risk of type 2 diabetes, particularly with the consumption of French fries. Namely, the risk of type 2 diabetes surged by 7.7% when the highest potato intake was compared to the lowest intake, but in case of French fries, that difference was 36.2%. Since French fries are a food item with a high AA level, acrylamide may arguably be one of the contributing factors in the prevalence of type 2 diabetes. Similarly, a large French prospective cohort study proposed a strong link between ultra-processed food intake and the risk of type 2 diabetes occurrence [54].

## 3. Effect of Acrylamide Treatment on Islets of Langerhans Beta-Cell Function

**Figure 2:**
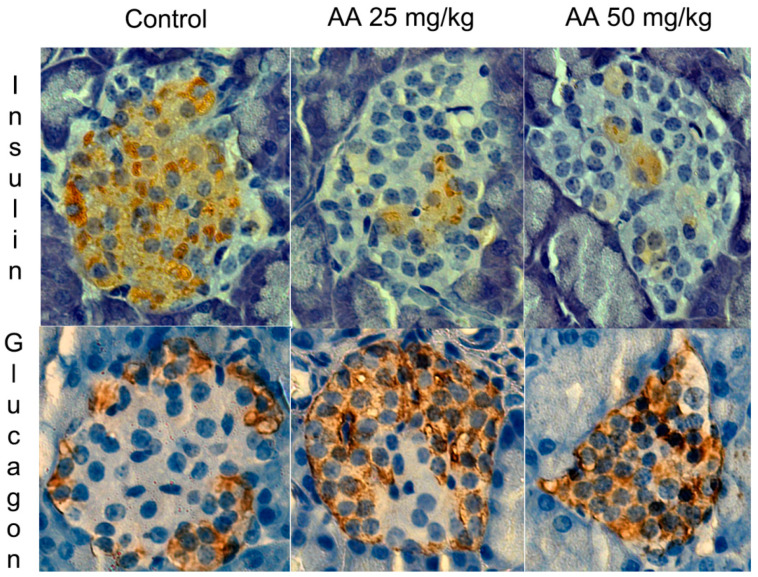
Insulin and glucagon *immunohistochemically stained* pancreatic islets of control and AA-treated rats. Male Wistar rats were treated with AA in doses of 25 mg/kg b.w./d and 50 mg/kg b.w./d; 400× light microscope magnification [58].

The islets of Langerhans are an endocrine component of the pancreas. They consist of five cell types (alpha, beta, delta, gamma and epsilon) that secrete hormones that regulate the metabolism of carbohydrates and lipids and control digestive system function. Most of the islets are made up of beta cells (~70%) placed in the center of the islets, while alpha (~20%) and other non-beta cells are distributed on the periphery (Figure 2) [55]. The critical role in maintaining glucose homeostasis in vivo in both animals and humans involves beta and alpha cells that secrete insulin and glucagon, respectively [56]. When the glucose concentration is high, insulin stimulates the uptake of glucose by most body cells from the blood stream in order to lower the level of glucose. Conversely, when the glucose concentration is low, glucagon promotes gluconeogenesis to elevate the level of glucose in the blood by increasing the breakdown of glycogen and the release of glucose by the liver [57].

**Figure 3:**
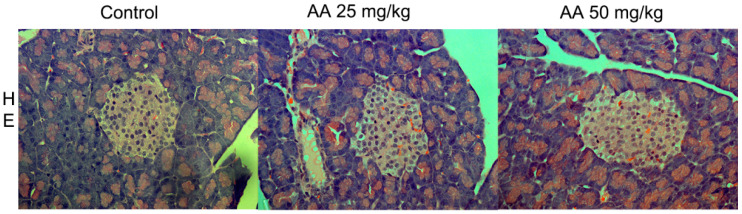
Hematoxylin and eosin (HE)-stained pancreatic islets of control and AA-treated rats. Male Wistar rats were treated with AA in doses of 25 mg/kg b.w./d and 50 mg/kg b.w./d; 400× light microscope magnification [58].

In our studies, we examined the effects of AA treatment in doses of 25 mg/kg b.w./d and 50 mg/kg b.w./d for three weeks on the endocrine pancreas of juvenile and adult male Wistar rats [58,59]. Irrespective of age, AA treatment did not affect blood glucose, insulin level or the histoarchitecture of islets of Langerhans, as illustrated in Figure 3 and Table 1. However, in both juvenile and adult male rats, pancreatic islet remodeling, determined by alpha-cell expansion and beta-cell mass reduction, was observed (Figure 2, Table 1). A similar composition of islets of Langerhans with a reduction in beta-cell number and an increase in alpha-cell mass was detected in rat and human diabetic pancreas [60,61]. Although alpha- and beta-cell remodeling upon AA exposure is not prominent as in diabetic pancreas, prolonged chronic exposure to AA could lead to decreases in the glucose and insulin levels in the blood as a consequence of progressed beta-cell mass reduction. Beta-cell reductions of 15% and 25% in male rats treated with AA in doses of 25 mg/kg b.w./d and 50 mg/kg b.w./d, respectively, did not impair normal levels of glucose and insulin in the blood [58,59]. Hyperglycemia appears when beta cells are reduced by 70–80%, as is observed in type 1 diabetes [62]. Normoglycemia in AA-treated male rats could be a result of the over-secretion of insulin from the remaining functional beta cells [58].

Our results showing no effect of AA treatment on the blood glucose level and histoarchitecture of islets in male rats [58,59] were later confirmed by Quan et al. [36], Karimani et al. [63], Alanazi et al. [64] and Zhao et al. [65]. Namely, an unchanged glucose level upon AA application was detected in adult male Wistar rats treated for two weeks with AA in doses of 20 mg/kg b.w./d [64] and 50 mg/kg b.w./d [63] and in adult male Balb/c mice treated with 50 mg/kg b.w./d of AA for one week [65]. In addition, Quan et al. [36] did not detect any histopathological changes in the islets of Langerhans of adult male Sprague–Dawley rats treated with acrylamide in a dose of 21 mg/kg b.w./d for six weeks.

Yue et al. [66] investigated the effects of AA exposure in doses of 15 mg/kg b.w./d and 30 mg/kg b.w./d for three weeks on the glucose homeostasis of adult female Sprague–Dawley rats. Contrary to the results obtained in male rats [36,58,59,63,64,65], Yue et al. [66] reported increased blood glucose and decreased plasma insulin levels as well as damaged islets in AA-treated female rats. The detected sex differences in the blood glucose and insulin levels and in the structure of islets could be a consequence of different rates of AA absorption [66]. Namely, there is a higher AA absorption rate in females compared to males [66,67]. Taken together, these data suggest that, concerning glucose homeostasis as well as the histopathological changes in the islets of Langerhans, female rats are more vulnerable to AA compared to male rats [36,58,59,63,64,66]. Sex-related responses in AA-exposed rats were also detected in hematological, biochemical and neurological parameters as well as in histopathological profiles of the liver, kidney, brain and testes [68].

Decreased insulin synthesis in the pancreas upon AA exposure was detected at both the mRNA [66] and protein levels [58,59]. Decreased insulin expression is a consequence of an AA-induced downregulation of pancreatic duodenal homeobox-1 (*pdx-1*) [66]. Pdx-1 is a transcriptional activator of the gene for insulin and has a pivotal role in beta-cell function and survival [69]. A partial loss of Pdx-1 causes severe beta-cell dysfunction and leads to beta-cell death and diabetes in rodents and humans [69].

## 4. Effect of Acrylamide Treatment on Oxidative Stress Parameters and CYP2E1 Expression in Pancreatic Beta Cells

Oxidative stress is a major mechanism of AA-induced toxicity in different organs [70,71]. Oxidative stress is an imbalance between pro-oxidants and antioxidants that develops as a consequence of increased production of reactive oxygen species (ROS) and/or reactive nitrogen species (RNS) and/or the depletion of the antioxidant defense system [72].

We examined whether exposure to AA disturbs the redox balance in pancreatic beta cells in in vitro and in vivo experimental models [72]. In vitro, pancreatic beta-cell line Rin-5F, a validated beta-cell model system, was treated with the half-maximal inhibitory concentration (IC_50_) of AA for 12 and 24 h. As an in vivo model system, we used islets of Langerhans of male Wistar rats treated subchronically with AA in doses of 25 mg/kg b.w./d and 50 mg/kg b.w./d for three weeks [72]. The exposure of the pancreatic beta-cell line to AA led to an increase in lipid peroxidation and GST activity and a decrease in GSH, while it had no effect on protein thiol (-SH) groups (Table 2) [72]. GSH is a major cellular reducing agent and antioxidant that, by interacting with ROS, protects cells [73]. Many xenobiotics, including AA, are detoxified by coupling with GSH [73]. AA is an alpha- and beta-unsaturated electrophile that interacts with cell nucleophiles with sulfhydryl, amino or hydroxyl reactive groups. During detoxification, most of the AA interacts with GSH, resulting in glutathione *S*-conjugate formation that consequently diminishes the GSH level [34,74]. GSH reduction to a critical level can induce lipid peroxidation [34], as observed in AA-treated Rin-5F cells (Table 2). Contrary to the GSH content that was diminished after AA application, protein thiol groups were not affected by AA (Table 2). Most of the disulfide-linked cytosolic proteins are enzymes that temporarily form disulfide bonds during the catalyzation of oxidation–reduction reactions [75]. In AA-exposed Rin-5F cells, reduced thioredoxin, a major cellular protein disulfide reductase, could bind to substrate proteins that contain disulfide bonds and reduce cysteine residues in order to avoid the oxidation of cytosolic cysteine [72,75]. The stimulation of GST activity in the AA-exposed pancreatic beta-cell line could be a result of an increased formation of S-conjugates between AA and GSH [34,72,74]. The reduced transcription of the GSTP1 gene in AA-exposed Rin-5F cells could be a consequence of downregulated Nrf2 expression (Table 2) since Nrf2 is a transcriptional activator of the GSP1 gene [72,76]. Furthermore, AA could alter the GST isozyme level in Rin-5F cells via the Kelch-like ECH-associated protein 1–nuclear factor erythroid 2-related factor 2–antioxidant response element (Keap1-Nrf2-ARE) signaling pathway [72,77].

**Figure 4:**
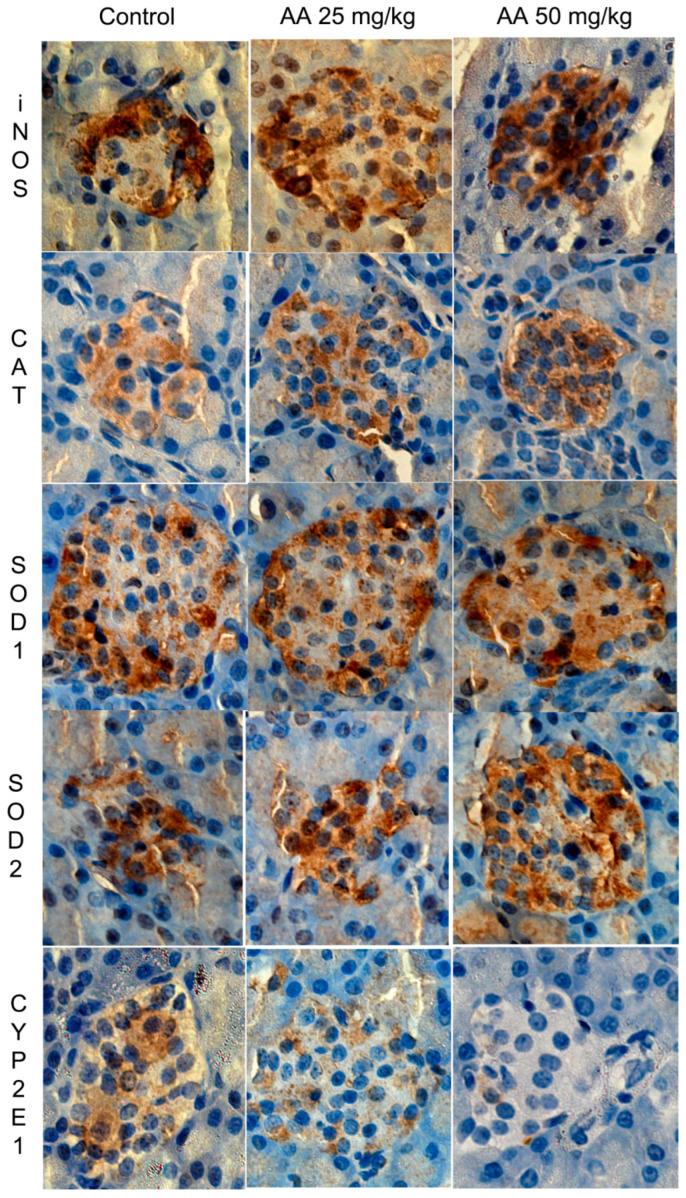
iNOS, CAT, SOD1, SOD2 and CYP2E1 *immunohistochemically stained* pancreatic islets of control and AA-treated rats. Male Wistar rats were treated with AA in doses of 25 mg/kg b.w./d and 50 mg/kg b.w./d; iNOS, inducible nitric oxide synthase; CAT, catalase; SOD, superoxide dismutase; CYP2E1, cytochrome P450 2E1; 400× light microscope magnification [72].

In our in vitro model, AA treatment decreased superoxide dismutase (SOD) and catalase (CAT) activity, increased SOD1 and SOD2 transcription and had no effect on CAT transcription in the pancreatic beta-cell line (Table 2) [72]. SOD and CAT play a pivotal role in maintaining cellular redox homeostasis [78]. SOD catalyzes the dismutation reaction of superoxide radicals to H_2_O_2_, which is then metabolized to H_2_O and O_2_ by CAT [79]. The reduced SOD enzymatic activity along with increased expression in AA-exposed Rin-5F cells may be due to the inactivation of excess protein that has been synthesized under conditions of high oxidative stress [80]. The inhibition of CAT activity could be a consequence of the elevated NO level in AA-treated Rin-5F cells (Table 2) [81]. Contrary to our in vitro findings, in the in vivo model AA exposure did not affect the levels of SOD1, SOD2 and CAT protein in rats’ islets of Langerhans (Figure 4, Table 1) [72]. A normal expression of antioxidant enzymes is essential for sustaining physiological amounts of ROS, a by-product of mitochondrial metabolism, within islets [82]. At a basal level, ROS act as signaling molecules for glucose-stimulated insulin secretion in beta cells [83]. In physiological conditions, ROS are involved in the regulation of islet gene expression and beta-cell function as well as in maintaining islet homeostasis [82]. A preserved expression of SOD and CAT in pancreatic islets upon AA application is particularly important for beta-cell function since beta cells have low antioxidant capacity, making them more vulnerable to oxidative stress [83]. The unaffected expression of antioxidant enzymes in islets could contribute to normal beta-cell functioning that is reflected in the normal serum insulin and glucose levels in AA-treated male rats (Table 1) [58,59,63,64,72].

**Figure 5:**
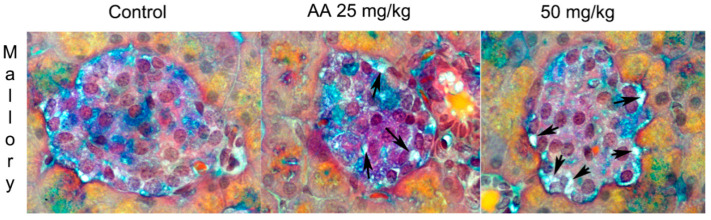
Mallory-Azan-stained pancreatic islets of control and AA-treated rats. Male Wistar rats were treated with AA in doses of 25 mg/kg b.w./d and 50 mg/kg b.w./d. Black arrows show dilated blood vessels; 400× light microscope magnification [72].

In our in vivo model, the induced *inducible nitric oxide synthase* (iNOS) expression upon AA application was observed in rats’ islets of Langerhans (Figure 4, Table 1) [72]. Nitric oxide (NO) is a free radical with a role as a signaling molecule that is necessary for normal islet physiology. iNOS is moderately expressed in healthy pancreatic islets [84]. However, increased iNOS expression leads to dysfunction in alpha and beta cells, inhibits insulin secretion and was detected in type 1 and type 2 diabetes and acute pancreatitis [85]. AA exposure strongly induced iNOS expression in almost all islet cells (Figure 4, Table 1) [72]. Increased iNOS could induce the vasodilatation of islet blood vessels (Figure 5) since nitric oxide is an endothelium-dependent vasodilator [72,86]. Islets are richly capillarized, and endocrine cells are surrounded by vascular endothelial cells [72]. In line with our in vivo results, increased activity and expression of iNOS were detected in the AA-treated pancreatic beta-cell line (Table 2) [72].

AA downregulated CYP2E1 in beta cells in both our in vitro (Table 2) and in vivo (Table 1, Figure 4) experimental models [72]. CYP2E1 is an enzyme that biotransforms AA to genotoxic epoxide GA. GA is more reactive than AA and can induce the formation of GA-DNA adducts [87,88]. The presence of CYP2E1 in pancreatic endocrine cells makes them capable of metabolizing AA to GA [89,90]. By expressing CYP2E1 in densely vascularized islets, endocrine cells are sensitive to xenobiotics, including AA, that enter the organism [90]. One possibility is that AA treatment may stimulate beta cells to decrease CYP2E1 expression (Figure 4, Table 1) to protect them from the formation of more toxic **glycidamide** [72]. A downregulation of CYP2E1 could be a protective response since CYP2E1 promotes oxidative stress, and a high level of the enzyme is toxic to cell [91]. The regulation of CYP2E1 by its own substrates has been reported previously, and it occurs at either the transcriptional or posttranscriptional level [92]. The other possibility is that AA-induced iNOS may bind to the iron–sulfur reaction center of CYP2E1 and destroy the protein, thus causing the downregulation of its expression [93]. The reduced CYP2E1 expression in AA-treated Rin-5F cells (Table 2) could cause the downregulation of Nrf2 transcription since an increase in CYP2E1 induces the expression of the Nrf2 gene [94]. Reduced CYP2E1 expression, along with the elevated GST activity and depletion of GSH content, suggests that AA is able to regulate its own metabolism in pancreatic beta cells [72].

In our in vivo study, the enhanced iNOS expression but unaffected expression of antioxidant enzymes may suggest a mild or moderate level of oxidative stress in the pancreatic islets of subchronically AA-treated rats. On the other hand, in the in vitro model system AA disturbed the majority of oxidative stress parameters and the activity of antioxidant enzymes in the pancreatic beta-cell line [73]. By inducing oxidative stress, AA may promote beta-cell dysfunction [95]. Furthermore, oxidative stress is implicated in beta-cell failure in type 1 diabetes [83].

## 5. Effect of Acrylamide Treatment on Glucose Metabolism and the Insulin Signaling Pathway

The liver has a critical role in the regulation of glucose homeostasis by controlling different pathways of glucose metabolism, including glycogenesis, glycogenolysis, glycolysis and gluconeogenesis [96]. The available results regarding the effects of AA on glucose metabolism are contradictory, reflecting sex and/or species differences [36,65,66]. Yue et al. [66] examined the effects of AA exposure on the expression of genes that code the proteins involved in glucose metabolism in the liver. Female Sprague–Dawley rats were treated with AA in doses of 15 mg/kg b.w./d and 30 mg/kg b.w./d for three weeks. AA led to an increase in the expression of two critical enzymes for gluconeogenesis: glucose-6-phosphatase (g6p) and pyruvate carboxylase (pc) in the livers of female rats [66,97,98,99]. G6p catalyzes the hydrolysis of glucose-6-phosphate produced in glycogenolysis and gluconeogenesis [97], while pc catalyzes the conversion of pyruvate to oxaloacetate [98,99]. The enhanced expression of these two enzymes upon AA application could indicate an increase in gluconeogenesis in female rats. In addition, female rats are exposed to an AA-induced upregulation of the main liver regulatory enzyme responsible for the control of blood glucose level: glycogen phosphorylase (gp) [66,100]. Gp catalyzes the initial step in glycogen degradation in the process of glycogenolysis [101]. An increased expression of gp is in accordance with the decreased liver glycogen content in AA-treated female rats [66]. The application of AA to female rats suppressed the expression of two critical enzymes in the glycolytic pathway: glucokinase (GCK) and 6-phosphofructokinase (PFK) [66,102,103,104,105]. GCK catalyzes the first reaction in glycolysis, the phosphorylation of glucose to glucose-6-phosphate, and acts as the pivotal glucose sensor in the organism that contributes to the maintenance of the blood glucose level [102,103]. PFK catalyzes the reaction of the phosphorylation of fructose-6-phosphate to fructose-1,6-bisphosphate, which represents the critical regulatory step in glycolysis [104,105]. The downregulation of these two enzymes could imply that AA inhibits the glycolytic pathway and thus impairs glucose homeostasis. The presented data showing the AA-induced stimulation of gluconeogenesis and glycogenolysis along with the inhibition of glycolysis are in agreement with the increased blood glucose level and diminished glycogen content in female rats [66].

Although the available data show that AA does not affect the blood glucose level in male rats and mice [58,59,63,64,65], Quan et al. [36] detected a decrease in two metabolites involved in the pentose phosphate pathway, gluconolactone and D-erythrose 4-phosphate (E4P), in male Sprague–Dawley rats treated with AA in a dose of 21 mg/kg b.w./d for six weeks. The pentose phosphate pathway is an important part of glucose metabolism that has been associated with type 2 diabetes mellitus [106]. Glucose-6-phosphate dehydrogenase deficiency is a metabolic disorder characterized by an altered level of gluconolactone and E4P [107]. Therefore, a reduction in these two metabolites suggests damaged liver glucose metabolism in male rats upon AA application [36,107,108]. However, whether the observed impaired liver glucose metabolism in male rats affects the blood glucose level it is not known since Quan et al. [28] did not determine the glucose concentration in the blood upon AA application.

Zhao et al. [65] reported a suppression of gluconeogenic genes (g6p and phosphoenolpyruvate carboxykinase—*pepck*) and unchanged blood glucose in male Balb/c mice treated AA in a dose of 50 mg/kg b.w./d for one week. The detected differences in glucose liver metabolism among female and male rats and male mice [36,65,66] could be sex-, species- or treatment-related. Females have a higher AA absorption rate than males [67], making females more susceptible to AA than males.

Impaired glucose metabolism was detected in an AA-exposed human osteosarcoma U_2_OS cell line [109]. AA treatment led to alterations in glycolysis/gluconeogenesis. Several glycolytic intermediates, including fructose-1,6 diphosphate, dihydroxyacetone phosphate, glycerate 3-phosphate, gluconate-6 phosphate, glycerate 2-phosphate and lactic acid, were decreased, indicating a reduced metabolic rate of glycolysis [109].

Insulin plays a pivotal role in the regulation of glucose uptake by metabolically active organs, such as the liver. The binding of insulin to its receptor activates the insulin receptor substrates/phosphatidylinositol 3-kinase/protein kinase B (IRS/PI3K/Akt) signaling pathway, which further stimulates the transport of glucose into hepatocytes by the glucose transporter (GLUT4) [110]. In the IRS/PI3K/Akt signaling cascade, the Akt-dependent phosphorylation of fork-headed box protein O1 (FoxO1) regulates glucose metabolism in the liver and thus sustains normoglycemia [111]. To the best of our knowledge, there are no data regarding the effects of AA on insulin signaling in males. On the other hand, data concerning the effects of AA treatment on the expression of genes that code the proteins involved in insulin signaling in the liver are available for females [66]. Namely, exposure to AA reduced the expression of genes for IRS1; PI3KCA, which encodes the p110 subunit of PI3K; Akt and FoxO1, while the expression of genes for the insulin receptor were not affected by AA in female rats, indicating that AA impairs components of the insulin signaling pathway downstream of the insulin receptor [66].

The literature data show that AA affects glucose metabolism and the insulin signaling pathway [65,66,109]. By impairing glucose metabolism, AA poses a potential risk to diabetes development, since diabetes mellitus is defined as a metabolic disorder with disturbed glucose metabolism and hyperglycemia [36].

## 6. Effect of Acrylamide Treatment on Diabetics

Diabetic complications develop as a consequence of oxidative stress and inflammation [112]. Diabetics are more sensitive to environmental toxicants than the general population [65,113,114]. Studies in rodent model systems have shown that AA exposure worsens histopathological, biochemical, oxidative stress and inflammatory biomarkers in diabetic rats and mice [37,63,64,65].

Karimani et al. [63] compared the effects of AA on histopathological and biochemical parameters in diabetic and non-diabetic rats. Diabetic and non-diabetic male Wistar rats were treated with 50 mg/kg of AA for two weeks. In diabetic rats, the exposure to AA led to an increase in creatinin, alanine aminotransferases, lactate dehydrogenase, blood urea nitrogen and the uric acid level in serum as well as an increase in the expression of CYP2E1 in the liver and kidney. In addition, the histopathological alterations in the kidney and liver were more severe in diabetic than in non-diabetic rats. The exacerbation of histopathological and biochemical parameters in AA-treated diabetic rats could be a consequence of increased CYP2E1 expression. Upregulated CYP2E1 could produce more toxic GA and thus cause more damage in the diabetic liver and kidney [63].

In the study of Alanazi et al. [64], differences in oxidative stress, biochemical and inflammatory biomarkers in AA-exposed diabetic and non-diabetic rats were detected. To diabetic and non-diabetic male Wistar rats, AA was applied in a dose of 20 mg/kg b.w./d for two weeks. AA exposure increased the levels of biochemical and inflammatory parameters in the serum of diabetic compared to non-diabetic rats. Namely, the levels of glucose, AST, ALT, ALP, cholesterol, urea, creatinine, IL-beta, IL-6 and TNF-alpha were elevated in the serum of AA-exposed diabetic rats compared to AA-treated non-diabetic rats. In addition, AA application further elevated lipid peroxidation and the NO level in the liver and kidney of diabetic rats as well as decreased the SOD activity and GSH content in the diabetic kidney [55]. Taken together, these results suggest that the administration of AA to diabetic rats worsens the metabolic profile, indicating that AA has greater deteriorating effects in the diabetic state compared to the non-diabetic state [64].

Zhao et al. [65] examined whether AA aggravates the disruption of glucose and lipid metabolism in diabetic mice. Diabetic male Balb/c mice were exposed to 50 mg/kg of AA for one week. Blood glucose levels were higher in AA-treated diabetic mice compared to non-treated diabetic mice, while the glycogen content was not changed, suggesting that the blood glucose level is controlled by a mechanism other than liver glycogen. In addition, AA exposure further downregulated the expression of pivotal regulatory genes for gluconeogenesis (g6p and *pepck*) in diabetic mice, indicating that AA exacerbates the disorder of glucose metabolism [65]. In addition to glucose metabolism, AA also disrupted the lipid status in the serum and liver of diabetic mice. Namely, AA treatment lowered the level of total triglycerides, total cholesterol and LDL- and HDL-cholesterol in serum as well as the total triglycerides and total cholesterol in the livers of diabetic mice. Furthermore, exposure to AA decreased the expression of genes involved in fatty acid synthesis (*fasn, scd1*, *srebp1, srebp2* and *hnf4α*), the transport of fatty acids and triglycerides (*cd36*), the β-*oxidaton of fatty acids (pparα, cpt1a* and *cyp7a1*) and the synthesis and transport of cholesterol (hmg-coar, *acat1* and *ldlr*). By affecting the expression of these four groups of genes, AA could lead to lipid metabolism dysfunction. Taken together, these data suggest that diabetes and AA treatment have interactive effects on disturbed glucose and lipid metabolism [65]. Moreover, AA treatment elevated the activity of cyclooxygenase-2 (COX-2) in diabetic mice [65]. COX is a pivotal enzyme that converts arachidonic acid to prostaglandins [115]. COX-2 is a highly inducible isoform of an enzyme at sites of inflammation [115]. Type 2 diabetes is associated with COX-related inflammation, which is characterized by increased prostaglandin production [116]. Chronic inflammation is an early phase in the pathogenesis of diabetes, while oxidative injury related to inflammation is a later phase [116,117]. Chronic liver inflammation results in disturbed glucose and lipid metabolism [118]. Therefore, enhanced inflammation, reflected by increased COX-2 activity in AA-treated diabetic mice, could contribute to glucose and lipid metabolism disorder [65]. AA exposure further enhanced lipid peroxidation and decreased the GSH level and antioxidase activities in diabetic mice, indicating that AA aggravates oxidative stress [65]. The Keap1- Nrf2/AREs *signaling pathway is responsible for the induction of the expression of genes with a role in antioxidant defense upon cellular exposure to environmental stress* [119,120]. Reduced SOD activity, along with a decreased expression of genes for NOS, *Nrf2* and *Keap1 in AA-exposed diabetic mice compared to non-treated diabetic mice, indicate that AA diminishes the antioxidant defense system as a result of oxidative stress, which consequently leads to glucose and lipid metabolism disturbances* [65]. A principal component analysis (PCA) of glucose and lipid metabolism and gene expression results suggests a relationship between abnormalities in glucose and lipid metabolism and AA exposure in diabetic mice [65]. PCA is an often-applied analytical method for dimensionality reduction in multidimensional data [65,121].

Quan et al. [37] analyzed the effects of AA on diabetes-associated cognitive dysfunction. Diabetes-associated cognitive dysfunction is a frequent diabetes complication, and approximately 60-70% of diabetics worldwide suffer from this complication [122,123,124,125]. In the study of Quan et al. [37], AA was applied in a dose of 1 mg/kg b.w./d to Goto–Kakizaki (GK) rats, a rat model of diabetes. Significantly worse diabetes-associated cognitive dysfunction in GK rats was proven by the novel object recognition and Y-maze tests [37]. In the brain tissue of AA-exposed GK rats, oxidative damage, neuroinflammation and metabolic disorders were detected [37]. Namely, AA exposure increased the ROS level and lipid peroxidation, while the GSH level, CAT and total antioxidant capacity were reduced, which consequently led to glial cell activation [37]. Moreover, AA treatment induced the release of cytokines involved in neuroinflammation, including interleukin-1beta, interleukin-6, tumor necrosis factor-alpha and lipopolysaccharide [37]. In addition, AA application impaired glucose, amino acid and energy metabolism pathways in the brain [37].

Taken together, these data show that AA exposure worsens the diabetic state in rats and mice via oxidative stress and inflammation, indicating that diabetic individuals could be more susceptible to AA toxicity than healthy individuals [37,63,64,65].

## 7. Conclusions

Several studies have revealed an association between AA exposure and biomarkers of diabetes in the human population [1,23,26,45]. In rodent model studies, AA led to a decrease in insulin expression via the pdx-1 transcription factor [66]. In male rats, AA promoted pancreatic islet remodeling, determined by alpha-cell expansion and beta-cell reduction [58]. Regarding glucose metabolism and pathological changes in pancreatic islets, female rats are more sensitive to AA than males, possibly due to a higher AA absorption rate in females [66]. Oxidative stress is a potential mechanism of AA-induced toxicity in beta cells in both in vitro and in vivo model systems [72]. Furthermore, animal and in vitro studies demonstrated that AA disturbs glucose metabolism and the insulin signaling pathway [36,65,66,109]. In addition, by inducing oxidative stress and inflammation, AA exacerbates the diabetic state in rodents, indicating that diabetics are more vulnerable to AA than healthy individuals [37,63,64,65]. Although AA is positively related to oxidative stress in pancreatic beta cells and endocrine disruption [26,65,72], the precise mechanism by which AA triggers the occurrence of diabetes mellitus remains undetermined and remains to be further studied.

## Figures and Tables

**Table 1:** Effect of AA application on relevant glucose metabolism and toxicity parameters of pancreatic islets in male rats. Adult male Wistar rats were treated with 25 mg/kg b.w./d, and 50 mg/kg b.w./d of AA for three weeks [58,62].

Measured Parameter	AA_25mg/kg_ vs. Control	AA_50mg/kg_ vs. Control
Glucagon immunopositivity	↑	↑
Insulin immunopositivity	↓	↓
iNOS immunopositivity	↑	↑
CAT immunopositivity	n.s.	n.s.
SOD1 immunopositivity	n.s.	n.s.
SOD2 immunopositivity	n.s.	n.s.
CYP2E1 immunopositivity	n.s.	↓
Serum glucose	n.s.	n.s.
Serum insulin	n.s.	n.s.

iNOS, inducible nitric oxide synthase; CAT, catalase; SOD, superoxide dismutase; CYP2E1, cytochrome P450 2E1. Increase (↑) and decrease (↓) represent statistically significant changes. n.s., not significant.

**Table 2:** Effect of AA treatment on toxicity parameters in pancreatic beta-cell line Rin-5F. Rin-5F cells were treated with IC_50_ AA for 12 and 24 h [72].

Measured Parameter	12 h AA Treatment vs. Control	24 h AA Treatment vs. Control
Lipid peroxidation	↑	↑
GSH	↓	↓
-SH groups	n.s.	n.s.
NO2	↑	↑
SOD activity	↓	↓
CAT activity	↓	n.s.
GST activity	n.s	↑
iNOS expression	↑	↑
SOD1 expression	↑	↑
SOD2 expression	↑	↑
CAT expression	n.s.	n.s.
GSTP1 expression	n.s.	↓
GSTA2 expression	n.s.	n.s.
CYP2E1 expression	↓	↓
Nrf2 expression	↓	↓

GSH, reduced gluthatione; -SH groups, protein thiol groups; NO_2_, nitrite; SOD, superoxide dismutase; CAT, catalase; GST, glutathione-S-transferase; iNOS, inducible nitric oxide synthase; CYP2E1, cytochrome P450 2E1; Nrf2, NF-E2 p45-related factor 2. Increase (↑) and decrease (↓) represent statistically significant changes. n.s., not significant.
